# The Staphylococcus aureus Cell Wall-Anchored Protein Clumping Factor A Is an Important T Cell Antigen

**DOI:** 10.1128/IAI.00549-17

**Published:** 2017-11-17

**Authors:** Keenan A. Lacey, John M. Leech, Stephen J. Lalor, Niamh McCormack, Joan A. Geoghegan, Rachel M. McLoughlin

**Affiliations:** a Host-Pathogen Interactions Group, School of Biochemistry and Immunology, Trinity Biomedical Sciences Institute, Trinity College Dublin, Dublin, Ireland; b Department of Microbiology, Moyne Institute of Preventive Medicine, School of Genetics and Microbiology, Trinity College Dublin, Dublin, Ireland; University of Illinois at Chicago

**Keywords:** Staphylococcus aureus, cell wall-anchored proteins, vaccine, clumping factor A, T helper cells, cellular immunity

## Abstract

Staphylococcus aureus has become increasingly resistant to antibiotics, and vaccines offer a potential solution to this epidemic of antimicrobial resistance. Targeting of specific T cell subsets is now considered crucial for next-generation anti-S. aureus vaccines; however, there is a paucity of information regarding T cell antigens of S. aureus. This study highlights the importance of cell wall-anchored proteins as human CD4^+^ T cell activators capable of driving antigen-specific Th1 and Th17 cell activation. Clumping factor A (ClfA), which contains N1, N2, and N3 binding domains, was found to be a potent human T cell activator. We further investigated which subdomains of ClfA were involved in T cell activation and found that the full-length ClfA N123 and N23 were potent Th1 and Th17 activators. Interestingly, the N1 subdomain was capable of exclusively activating Th1 cells. Furthermore, when these subdomains were used in a model vaccine, N23 and N1 offered Th1- and Th17-mediated systemic protection in mice upon intraperitoneal challenge. Overall, however, full-length ClfA N123 is required for maximal protection both locally and systemically.

## INTRODUCTION

Staphylococcus aureus is a significant opportunistic pathogen that causes serious invasive infections in community and health care settings (1). Shockingly, resistance to every antistaphylococcal agent licensed to date has arisen (2). Thus, there is an urgent need to develop alternative treatments, and vaccines that prevent or lessen the severity of S. aureus infections may offer a potential solution; however, despite substantial efforts, no such vaccine has been licensed as yet (3).

A number of vaccines that induced neutralizing and opsonizing antibodies were effective in preclinical models (4); however, they did not confer protection in clinical trials, despite inducing robust humoral immunity (3). It is now widely accepted that T cells, in particular T helper (Th) cells, play a crucial role in protection against S. aureus infection. Th1 and Th17 cells have been shown to be critical for controlling phagocytic cell responses and thus enabling bacterial clearance during systemic and cutaneous infections (5–7).

Targeting individual T cell subsets is now considered an important strategy for next-generation anti-S. aureus vaccines (8). However, to date, no well-established S. aureus T cell epitopes have been identified. It has recently been shown that the majority of adults possess significant levels of circulating antigen-specific memory T cells, indicative of their previous exposure to S. aureus through either commensal colonization or previous subclinical infection (9). Kolata et al. demonstrated that extracellular proteins, composed of both secreted proteins and surface-bound proteins, elicited greater T cell responses than intracellular proteins (9). We have further demonstrated that heat-inactivated S. aureus, washed to remove all secreted factors, elicits a robust T cell response, suggesting surface-located factors of S. aureus are important for T cell activation (5).

S. aureus can express up to 25 different cell wall-anchored (CWA) proteins, which are covalently bound to the cell wall peptidoglycan by transpeptidases known as sortases (10, 11). Many CWA proteins are multifunctional and are involved in adhesion, invasion, biofilm formation, and/or evasion of host immune responses (11). However, for the most part, there is a lack of understanding of how these CWA proteins interact with immune pathways, and their capacity to activate T cells remains to be fully established. The CWA protein clumping factor A (ClfA) mediates binding to fibrinogen and fibrin and is considered a good vaccine candidate because it is expressed by the majority of strains and is a major virulence factor contributing to pathogenesis (12–14). ClfA contains an N-terminal ligand-binding A domain composed of three subdomains, N1, N2, and N3. The N23 subdomains are involved in binding to fibrinogen using the “dock, lock, and latch” mechanism (15). Ten residues located at the junction between the N1 and N2 subdomains are required for protein export and cell wall localization (16). However, a role for the remainder of the N1 subdomain remains elusive.

In the present study, we investigated the abilities of the CWA proteins to activate human T cells. As ClfA was a potent T cell activator, we further investigated the individual subdomains of ClfA as T cell antigens and demonstrated that the N23 and N1 subdomains individually could drive Th1 expansion in human T cells comparable to that of the full-length ClfA protein; however, only N23 was required for maximal Th17 cell expansion. Furthermore, when used in a model vaccine, N23 and N1 offered Th1- and Th17-mediated protection in mice upon systemic challenge similar to that of the full-length protein.

## RESULTS

### Staphylococcal cell wall-anchored proteins drive antigen-specific responses in human CD4^+^ T cells.

**FIG 1:**
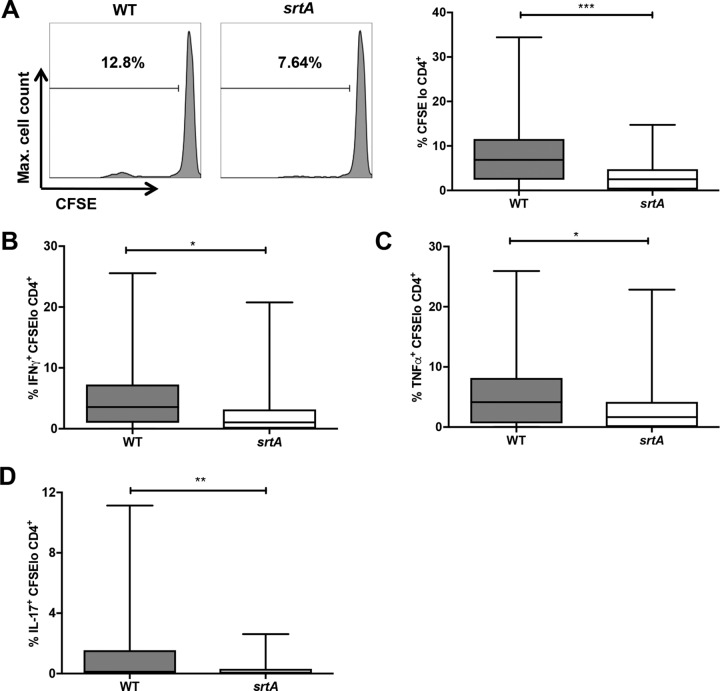
Human CD4^+^ T cells show greater antigen-specific responses to S. aureus LAC::*lux* WT than to LAC::*lux srtA* (*srtA*). (A) Purified CD4^+^ T cells were CFSE labeled and cocultured with autologous irradiated APCs from healthy blood donors and incubated with heat-inactivated S. aureus LAC::*lux* WT or LAC::*lux srtA* (1 μg/ml) or medium alone. On day 10, proliferation was assessed by gating on CFSE^lo^ cells in the CD4^+^ population. Representative fluorescence-activated cell sorter (FACS) plots of proliferating CD4^+^ cells are shown. (B to D) S. aureus-specific IFN-γ (B), TNF-α (C), and IL-17 (D) responses were assessed by gating on IFN-γ^+^, TNF-α^+^, or IL-17^+^ cells within the CFSE^lo^ CD4^+^ population. For each donor, medium-only responses were subtracted from responses to heat-inactivated S. aureus to determine the antigen-specific response. The results are shown as box-and-whiskers plots, where the horizontal line indicates the median, the boundaries of the box represent the interquartile range (IQR), and the whiskers indicate the highest and lowest values of the results. *n* = 34 to 40 per group. A nonparametric Mann-Whitney U test was used to analyze variances between groups. *, *P* < 0.05; **, *P* < 0.01; ***, *P* < 0.001.

To confirm that CWA proteins have the capacity for human T cell activation, the heat-inactivated LAC::*lux* wild-type (WT) strain or the LAC::*lux srtA* mutant strain, which lacks all surface-bound CWA proteins, was incubated with CD4^+^ T cells isolated from healthy adults and autologous irradiated antigen-presenting cells (APCs). Proliferation and cytokine production were assessed on day 10, and responses to medium alone were subtracted from responses to heat-inactivated S. aureus. The proportion of antigen-specific proliferating CD4^+^ T cells was significantly elevated in cells stimulated with the WT strain compared to cells stimulated with the *srtA* mutant (Fig. 1A). There was a wide spread in the proportion of CD4^+^ T cells proliferating in response to heat-inactivated S. aureus. The WT strain induced proliferation in 90% of individuals compared to the *srtA* mutant, which induced proliferation in only 77% of individuals (see Table S1 in the supplemental material). The proportions of CD4^+^ T cells showing both proliferation and production of gamma interferon (IFN-γ) (Fig. 1B), tumor necrosis factor alpha (TNF-α) (Fig. 1C), and interleukin 17 (IL-17) (Fig. 1D) were significantly higher in WT-stimulated cells than in *srtA* mutant-stimulated cells. This suggests that CWA proteins are capable of driving Th1 and Th17 cell expansion.

**FIG 2:**
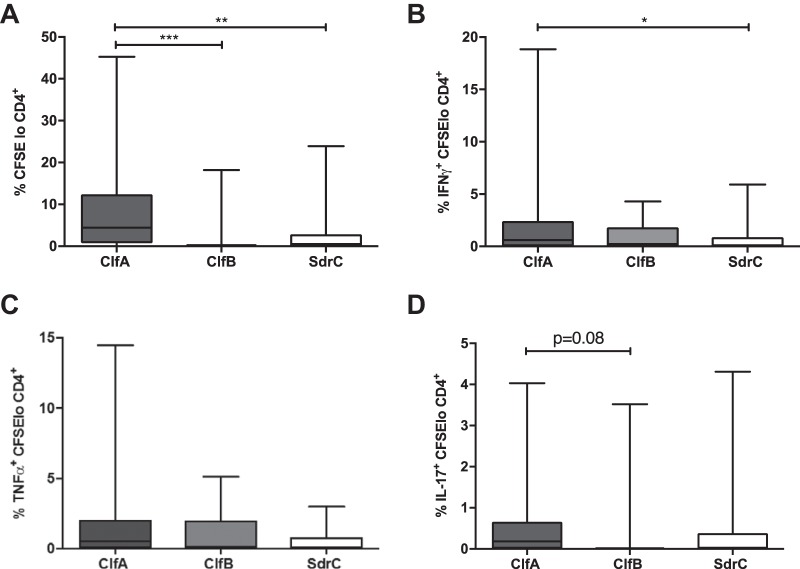
Human CD4^+^ T cells proliferate in response to purified staphylococcal cell wall-anchored proteins. (A) Purified CD4^+^ T cells were CFSE labeled and cocultured with autologous irradiated APCs from healthy blood donors and with ClfA, ClfB, SdrC (0.88 μM), or medium alone. On day 10, proliferation was assessed by gating on CFSE^lo^ cells in the CD4^+^ population. (B to D) S. aureus-specific IFN-γ (B), TNF-α (C), and IL-17 (D) responses were assessed by gating on IFN-γ^+^, TNF-α^+^, or IL-17^+^ cells within the CFSE^lo^ CD4^+^ population. For each patient, medium-only responses were subtracted from responses to purified S. aureus proteins to determine the antigen-specific response. The results are shown as box-and-whiskers plots, where the horizontal line indicates the median, the boundaries of the box represent the IQR, and the whiskers indicate the highest and lowest values of the results. *n* = 22 to 39 per group. A Kruskal-Wallis test with Dunn's multiple-comparison posttest was used to compare variances between groups. *, *P* < 0.05; **, *P* < 0.01; ***, *P* < 0.001.

The abilities of selected staphylococcal CWA proteins, ClfA, clumping factor B (ClfB), and serine aspartate repeat protein C (SdrC), to activate CD4^+^ T cell proliferation were then investigated. CD4^+^ T cells were cocultured with irradiated APCs and stimulated with purified proteins. Antigen-specific proliferation and cytokine production were assessed on day 10, and responses to medium alone were deducted. Of note, no difference in CD4^+^ T cell proliferation following stimulation with medium alone or an irrelevant control antigen (bovine serum albumin) was observed (see Fig. S1 in the supplemental material). Seventy-three percent of individuals had CD4^+^ T cells capable of responding to ClfA, while 58% of individuals responded to SdrC and 40% responded to ClfB (see Table S1 in the supplemental material). The levels of CD4^+^ T cell proliferation were significantly higher in ClfA-stimulated cells than in cells stimulated with ClfB or SdrC (Fig. 2A). All three of the proteins had the ability to induce the production of IFN-γ (Fig. 2B), TNF-α (Fig. 2C), and IL-17 (Fig. 2D) from CD4^+^ T cells, with a trend toward ClfA inducing a greater response than ClfB and SdrC. These results indicate that CWA proteins have the potential to activate T cells; however, some proteins, such as ClfA, may be more potent than others.

### Subdomains of ClfA drive antigen-specific responses in human CD4^+^ T cells.

To further probe the use of ClfA as a T cell antigen, we investigated which subdomains of the protein might be involved in T cell activation. Full-length ClfA A domain (ClfA N123), alongside two truncated proteins, ClfA N23 and ClfA N1, was used in the assay. Subdomains N2 and N3 were expressed as a single polypeptide, since together they represent a functional subunit, the minimum fibrinogen binding region. ClfA N1 was highly unstable and prone to degradation. To increase the protein's stability, it was bound to glutathione *S*-transferase (GST). The stabilities of these proteins were assessed (see Fig. S2 in the supplemental material). To account for the presence of GST, purified GST was also used to treat cells in the assay, and the results for N1 were corrected by subtracting any proliferation or cytokine production caused by GST alone. Only T cells from individuals who could respond to full-length ClfA N123 (i.e., the donors had ClfA-specific T cells) (see Fig. S3 in the supplemental material) were selected to accurately assess the subdomains involved in T cell activation.

**FIG 3:**
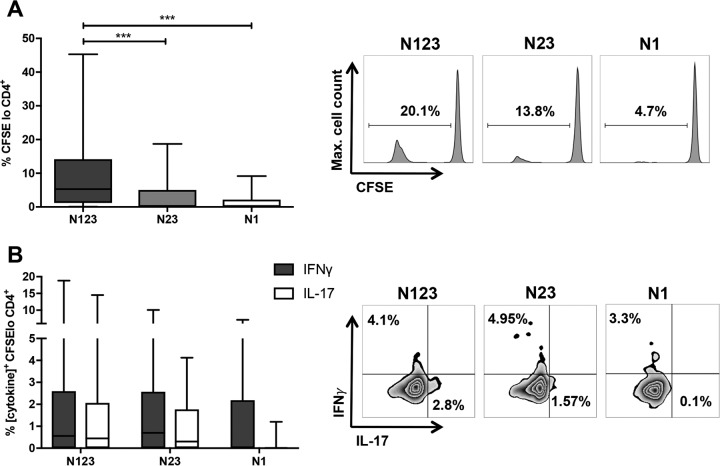
Human CD4^+^ T cells proliferate in response to subdomains of ClfA. (A) Purified CD4^+^ T cells were CFSE labeled and cocultured with autologous irradiated APCs from healthy blood donors and with ClfA N123, N23, or N1 (0.88 μM) or medium alone. On day 10, proliferation was assessed by gating on CFSE^lo^ cells in the CD4^+^ population. A Kruskal-Wallis test with Dunn's multiple-comparison posttest was used to compare variances between groups. ***, *P* < 0.001. (B) S. aureus antigen-specific Th1 and Th17 proportions were compared by gating on IFN-γ^+^ or IL-17^+^ cells within in the CFSE^lo^ CD4^+^ population. For each donor, medium-only responses were subtracted from responses to purified protein to determine the antigen-specific response. The results are shown as box-and-whiskers plots, where the horizontal line indicates the median, the boundaries of the box represent the IQR, and the whiskers indicate the highest and lowest values of the results. Representative FACS plots of CD4^+^ cells are shown. *n* = 20 to 30 per group.

The percentage of CD4^+^ T cells dividing in response to N123 was significantly greater than for cells treated with either the N23 or N1 subdomain (Fig. 3A). All three subdomains were capable of activating Th1 cells to similar extents (Fig. 3B). In contrast, however, it appeared that the N1 domain was not required for maximal Th17 cell expansion (Fig. 3B). Full-length N123 and N23 domains induced almost equivalent Th17 cell expansion. These results suggest that the N23 domain possesses both Th1 and Th17 cell-activating abilities; however, the N1 domain can potentially activate only Th1 cells.

### Immunization with individual subdomains of ClfA induces cellular and humoral responses.

Having confirmed that ClfA N123, N23, and N1 can promote human Th1 and Th17 responses *in vitro*, albeit to differing extents, we investigated if these domains could promote Th1- and Th17-mediated protection against S. aureus systemic infection when used in a model vaccine. We utilized a previously described model S. aureus vaccine (5) composed of ClfA N123 or its individual subdomains formulated with the Toll-like receptor 9 (TLR9) agonist CpG as an adjuvant. Groups of naive mice were vaccinated with CpG alone, CpG plus N123, CpG plus N23, CpG plus N1, or phosphate-buffered saline (PBS) alone and then challenged on day 63 before assessing infection outcomes and protective immune mechanisms. To account for the presence of GST-bound N1, mice were vaccinated with CpG plus GST. There was no difference between GST-vaccinated mice and PBS- and CpG-vaccinated mice (data not shown).

**FIG 4:**
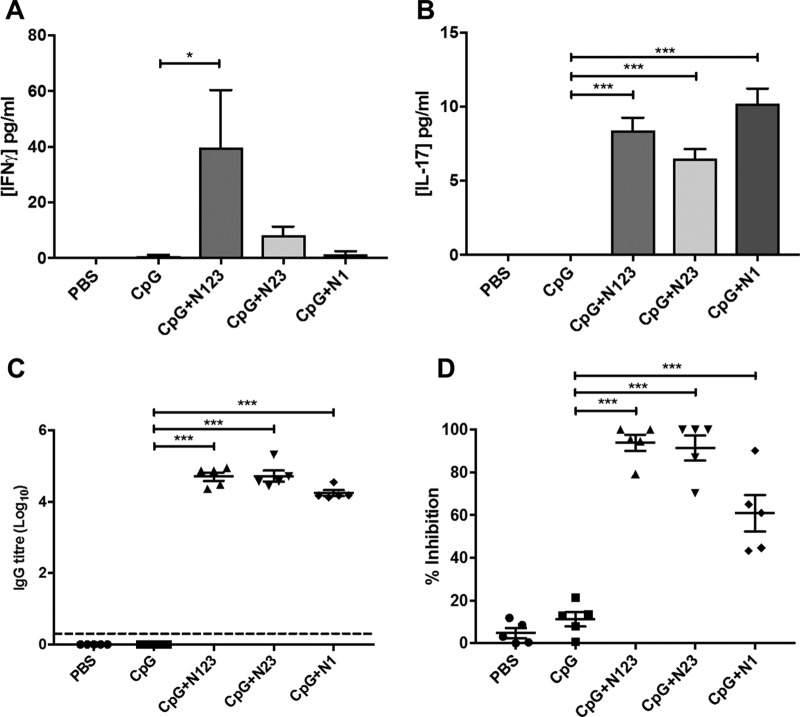
Immunization with individual subdomains of ClfA induces ClfA-specific cellular and humoral responses. (A and B) Mice were vaccinated with CpG (50 μg/mouse) plus ClfA N123, N23, or N1 (1 μg/mouse) via s.c. injection on days 0, 14, and 28. Antigen-specific responses by cells isolated from the inguinal lymph nodes were determined on day 63 by *ex vivo* stimulation with medium or ClfA N123 (5 μg/ml) for 72 h and subsequent ELISA to determine levels of IFN-γ (A) and IL-17 (B). Medium-only responses were subtracted from responses to ClfA N123. (C) On day 63, sera were collected from vaccinated mice. ClfA N123-specific IgG titers were determined using ELISA. The dashed line indicates the limit of detection (log_10_ 0.301). (D) Neutralizing antibodies were determined by measuring the ability of serum (1:60 dilution) to inhibit S. aureus PS80 adherence to fibrinogen via ClfA. The results are expressed as means and standard errors of the mean (SEM). *n* = 5 per group. One-way ANOVA with a Bonferroni posttest was performed to compare variances between CpG and vaccinated groups. *, *P* < 0.05; ***, *P* < 0.001.

Antigen-specific cellular and humoral responses were assessed prior to challenge. Immunization with CpG plus N123 drove a significant increase in IFN-γ production by inguinal lymph node (ILN) cells compared to CpG-immunized mice following 72-h *in vitro* restimulation with ClfA N123, whereas vaccination with CpG plus N23 and CpG plus N1 resulted in a modest increase (Fig. 4A). IL-17 production by ILN cells was significantly increased in all vaccinated groups compared to those vaccinated with CpG alone (Fig. 4B); however, the levels of IL-17 were low compared to the IFN-γ response, consistent with the use of the Th1-inducing adjuvant CpG. Anti-ClfA N123 IgG titers were significantly elevated in the sera of all immunized mice compared to those that received adjuvant alone (Fig. 4C). Neutralizing antibodies were also present in the sera of all immunized mice and effectively inhibited the binding of S. aureus PS80 to fibrinogen via ClfA (Fig. 4D).

### Immunization with individual subdomains of ClfA protects against S. aureus systemic infection.

**FIG 5:**
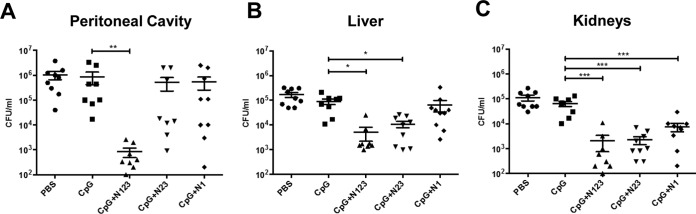
Immunization with individual subdomains of ClfA protects against S. aureus infection. Mice were vaccinated with CpG (50 μg/mouse) plus ClfA N123, N23, or N1 (1 μg/mouse) via s.c. injection on days 0, 14, and 28. On day 63, the mice were challenged with S. aureus PS80 (5 × 10^8^ CFU) via i.p. injection alongside a control group of sham (PBS)-immunized mice. At 72 h postinfection, the bacterial burden was assessed in the peritoneal cavity (A), liver (B), and kidneys (C). The results are expressed as log_10_ CFU per milliliter, with means indicated by the horizontal lines. The data are pooled from the results of 2 independent experiments; *n* = 8 to 10 per group. One-way ANOVA with a Bonferroni posttest was performed to compare variances between CpG and vaccinated groups. *, *P* < 0.05; **, *P* < 0.01; ***, *P* < 0.001.

Vaccination with CpG plus N123 significantly enhanced the clearance of S. aureus infection from the peritoneal infection site (∼3-log_10_-unit reduction) at 72 h postinfection (Fig. 5A) compared to the CpG-alone group. We had previously established 72 h as the peak of infection in this model (5, 17). Vaccination with N23 or N1 did not reduce the bacterial burden in the peritoneal cavity. Interestingly, vaccination with CpG plus N23 conferred systemic protection at levels comparable to those with full-length ClfA N123 by significantly reducing the bacterial burden in the liver (∼1 log_10_ unit) (Fig. 5B) and the kidneys (∼1.4 log_10_ units) (Fig. 5C). However, vaccination with CpG plus N1 conferred significant systemic protection only in the kidneys (∼1.0 log_10_ unit) (Fig. 5C).

**FIG 6:**
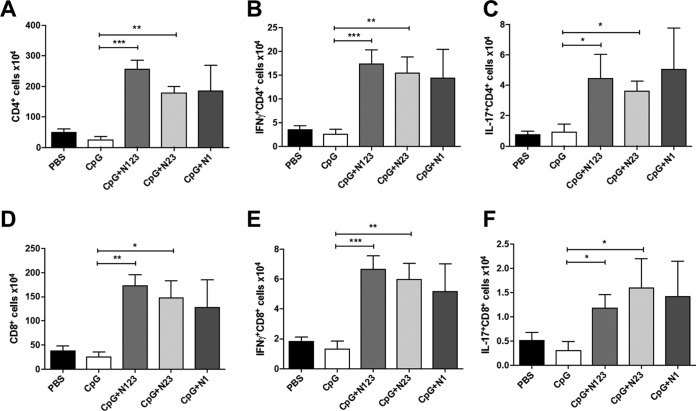
Immunization with individual subdomains of ClfA plus CpG increases CD4^+^ and CD8^+^ T cell recruitment and activation at the site of infection during S. aureus infection. (A and D) Mice were vaccinated with CpG (50 μg/mouse) plus ClfA N123, N23, or N1 (1 μg/mouse) via s.c. injection on days 0, 14, and 28. On day 63, the mice were challenged with S. aureus PS80 (5 × 10^8^ CFU) via i.p. injection alongside a control group of sham (PBS)-immunized mice. At 72 h postinfection, cells were isolated from the peritoneal cavity to assess absolute numbers of CD4^+^ (A) and CD8^+^ (D) T cells. (B, C, E, and F) The numbers of IFN-γ^+^ CD4^+^ (B), IL-17^+^ CD4^+^ (C), IFN-γ^+^ CD8^+^ (E), and IL-17^+^ CD8^+^ (F) cells in the peritoneum were assessed by flow cytometry. The results are expressed as means and SEM. The data are pooled from the results of 2 independent experiments; *n* = 8 to 10 per group. One-way ANOVA with a Bonferroni posttest was performed to compare variances between CpG and vaccinated groups. *, *P* < 0.05; **, *P* < 0.01; ***, *P* < 0.001.

To determine if the vaccine-induced protection was associated with an enhanced T cell response, the cells infiltrating the peritoneal cavity at 72 h postinfection were assessed. The total number of infiltrating CD4^+^ T cells was elevated in all the vaccinated groups compared to the control groups (Fig. 6A). The number of CD4^+^ T cells producing IFN-γ (Fig. 6B) and IL-17 (Fig. 6C) was also increased in the peritoneal cavities of CpG- plus N123-, CpG- plus N23-, and CpG- plus N1-immunized mice compared to control groups. There were also elevated levels of CD8^+^ cells infiltrating the peritoneal cavity in all groups compared to control animals (Fig. 6D). IFN-γ (Fig. 6E) and IL-17 (Fig. 6F) production by these CD8^+^ cells was increased in all vaccinated groups compared to controls.

Overall, these results reveal that vaccination with full-length N123, N23, or N1 in combination with the adjuvant CpG increased IFN-γ and IL-17 production by both CD4^+^ and CD8^+^ T cells at the site of infection. Of note, vaccination with either full-length N123 or subdomain N23 or N1 in combination with CpG had no significant effect on the production of IFN-γ or IL-17 by γδ T cells at this time point (see Fig. S4 in the supplemental material).

**FIG 7:**
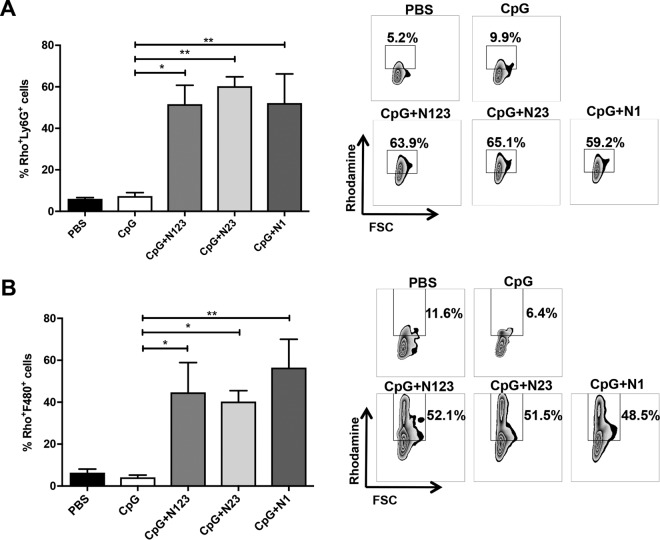
Immunization with individual subdomains of ClfA enhances phagocyte ROS activity during S. aureus infection. Mice were vaccinated with CpG (50 μg/mouse) plus ClfA N123, N23, or N1 (1 μg/mouse) via s.c. injection on days 0, 14, and 28. On day 63, the mice were challenged with S. aureus PS80 (5 × 10^8^ CFU) via i.p. injection alongside a control group of sham (PBS)-immunized mice. At 72 h postinfection, neutrophils (Ly6G^+^ F480^−^) (A) and macrophages (F480^+^ Ly6G^−^) (B) were assessed for ROS activity, detected by rhodamine (Rho)-positive cells. The results are expressed as means and SEM, and representative FACS plots of neutrophils (A) and macrophages (B) are shown. *n* = 5 per group. One-way ANOVA with a Bonferroni posttest was performed to compare variances between CpG and vaccinated groups. *, *P* < 0.05; **, *P* < 0.01.

Finally, we established the downstream effects of this ClfA-induced protective Th1 and Th17 immunity. Vaccination with subdomains of ClfA in combination with CpG did not affect the total numbers of phagocytes recruited to the peritoneal cavity during S. aureus infection (see Fig. S5 in the supplemental material). However, the phagocytes present in vaccinated mice displayed increased activation, as assessed by reactive oxygen species (ROS) activity. Significantly elevated ROS activity was observed in neutrophils (Fig. 7A) and macrophages (Fig. 7B) in all the vaccinated groups compared to CpG controls at 72 h postinfection. Taken together, these results demonstrate that vaccination with full-length N123, N23, or N1 can drive Th1 and Th17 responses, which in turn enhance neutrophil and macrophage effector functions to prevent S. aureus systemic dissemination.

## DISCUSSION

Targeting T cell subsets is now considered an important strategy for next-generation anti-S. aureus vaccines, and it is vital that we include relevant components to activate appropriate immune responses in future anti-S. aureus vaccines. Phase I/Ib clinical trials of NDV-3A by NovaDigm (18) and phase I trials of four-component S. aureus vaccine by GlaxoSmithKline (GSK) (19) have demonstrated T cell responses and associated cytokine production in vaccinated individuals; however, in the case of the GSK vaccine, T cell responses were very low (19). The NDV-3A vaccine is composed of a single antigen that is structurally similar to ClfA (18), while the GSK anti-S. aureus vaccine is multivalent, composed of ClfA N123, capsular polysaccharide types 5 and 8, and alpha toxin (19). Surface-exposed capsular polysaccharide and wall teichoic acid of S. aureus have previously been shown to activate T cells (20–23); however, S. aureus CWA protein T cell epitopes have yet to be identified. This study highlights the importance of CWA proteins as T cell activators specifically capable of driving Th1 and Th17 immune responses *in vivo* but also, crucially, using human *in vitro* T cell cultures.

The healthy population possesses on average 3.6% circulating S. aureus-specific T cells, indicative of an individual's prior exposure to S. aureus through commensal colonization or previous subclinical infections (9). This means that human blood from healthy volunteers can be used to screen antigens for their human T cell-activating capabilities. An approach such as this could be utilized in advance of clinical trials to direct us toward specific antigens that may be useful T cell activators. Using this approach, we found that heat-inactivated S. aureus (which contains a pool of S. aureus cell surface-associated antigens) was a potent human CD4^+^ T cell activator; however, when CWA proteins were absent in the SrtA-deficient mutant, CD4^+^ T cell activation (proliferation and cytokine production) was significantly reduced. Subsequently, we demonstrated that purified ClfA, ClfB, and SdrC were all capable of driving antigen-specific responses in human CD4^+^ T cells and had the ability to induce significant levels of both Th1 and Th17 cell activation. Although ClfA was the most potent T cell activator, all the proteins were capable of inducing responses, highlighting the fact that CWA proteins represent an important pool of T cell antigens. We further investigated which subdomains of ClfA were involved in T cell activation and found that the full-length ClfA N123 and N23 were potent Th1 and Th17 activators. Interestingly the N1 subdomain was capable of activating Th1 cells; however, it was not a potent Th17 activator. This suggests that the Th17-activating region of ClfA is located within the N23 domain, whereas there is redundancy in the part of ClfA that can activate Th1 cells. Importantly, this work highlights the need for a systematic screen of all staphylococcal CWA proteins to identify the most potent T cell activators and, furthermore, indicates that unique epitopes may be present in such molecules, which are recognized by distinct T cell subsets.

To investigate whether subdomains of ClfA have differing capabilities to activate T cells *in vivo*, we produced a model antistaphylococcal vaccine combining either ClfA N123, N23, or N1 with the adjuvant CpG to investigate how the N23 and N1 subdomains would compare to the full-length ClfA N123 protein as vaccine antigens. Adjuvants play an important role in directing the nature of the immune response elicited by a vaccine, and distinct T cell subsets may be targeted by careful adjuvant selection. CpG is a TLR9 agonist, which is a potent inducer of Th1 responses (24). When alum was replaced with CpG in the acellular pertussis vaccine, it drove an antigen-specific Th1/Th17 response instead of a Th2/Th17 response, leading to greater protection against pertussis challenge (25). In a recent study, vaccination with ClfA N23 combined with alum did not induce a Th1 or Th17 response in mice (26); however, when sigma adjuvant system (SAS) was added to the adjuvant, it did elicit both a Th1 and a Th17 response. Despite this however the vaccine strategy used in the study failed to confer protection in the specific infection models used.

In our hands, vaccination with CpG plus N123 induced significant levels of IFN-γ and IL-17 from ILN cells upon *ex vivo* restimulation, and this was associated with protection upon systemic challenge. In contrast to the human studies, vaccination of mice with CpG plus N23 and CpG plus N1 induced low levels of IFN-γ. Low levels of IL-17 were detected in CpG- plus N23- and CpG- plus N1-vaccinated mice; however, they were significantly elevated above those of mice that received adjuvant alone, highlighting the fact that the N1 domain may be capable of inducing IL-17 activation in mice but not in humans. Importantly all three vaccine antigens were capable of conferring protection upon systemic challenge. Vaccination with CpG plus N123 offered protection locally at the site of infection and in the peripheral organs (liver and kidneys), which complements our previous findings (5). Vaccination with the subdomains N23 and N1 caused only a modest reduction in the bacterial burden at the peritoneal infection site. Bacterial dissemination to the kidneys in groups vaccinated with N23 and N1 was reduced to levels similar to those in ClfA N123-vaccinated mice. If translated to humans, even a 1-log-unit reduction in the bacterial burden in peripheral organs would likely significantly improve the prognosis of S. aureus systemic infection. This protection against systemic dissemination appears to be associated with increased T cell responses, as the levels of IFN-γ- and IL-17-producing CD4^+^ and CD8^+^ cells were elevated in all vaccinated groups postinfection, with a resultant increase in downstream phagocyte responses.

Previously, the only role attributed to the N1 subdomain of ClfA was that of 10 residues known to be crucial for export and cell wall localization of ClfA (16). For the first time, this study demonstrates that the N1 subdomain contains T cell epitopes capable of activating Th1 cells. Interestingly, antibodies from CpG- plus N1-vaccinated mice were also capable of inhibiting binding to fibrinogen, despite the fact that the N1 domain is not involved in fibrinogen binding. This inhibition of binding may be due to N1 antibodies sterically blocking the binding of ClfA to fibrinogen. Importantly, these results support the inclusion of the full-length ClfA N123 protein as an antigen in multicomponent anti-S. aureus vaccines, since the combination of N1, N2, and N3 provides the most robust response in human T cells and the best protection in our murine vaccination model.

It is now widely accepted that an effective anti-S. aureus vaccine should have three main attributes: (i) the ability to induce a robust T cell response to promote phagocyte recruitment and effective bacterial clearance, (ii) induction of antibodies to neutralize CWA proteins, and (iii) induction of antibodies to neutralize toxins. Previous studies have questioned the usefulness of ClfA as a vaccine antigen (26). However, this study fully supports the use of CWA proteins as vaccine antigens, as they have the ability to induce neutralizing antibodies but, more importantly, are capable of driving a much-needed cellular response. We have demonstrated that ClfA has the capacity to induce robust T cell responses and that the full-length ClfA N123 protein is required for maximum Th1 and Th17 activation *in vitro* and *in vivo*.

## MATERIALS AND METHODS

### Mice.

Age (6 to 8 weeks)- and sex-matched wild-type C57BL/6 mice were housed under specific-pathogen-free conditions at the Trinity College Dublin Comparative Medicines unit. All experiments were conducted in accordance with the recommendations and guidelines of the health product regulatory authority, the competent authority in Ireland, and with protocols approved by the Trinity College Dublin Animal Research Ethics Committee.

### Bacterial strains and growth conditions.

S. aureus strains USA300 LAC::*lux* WT (27) and PS80 (22) have been described previously. Consistent with ongoing work in our laboratory, LAC::*lux* was selected as the parental wild-type strain, and we confirmed that it performed the same as LAC (28) in our assay system (see Fig. S6 in the supplemental material). LAC::*lux srtA*::Erm^r^ (*srtA*) was constructed by the transduction of *srtA*::Erm^r^ using bacteriophage 85 (29). All bacteria were grown at 37°C for 24 h on tryptic soy agar (TSA). Bacteria were heat inactivated for the human assays, as previously described (5). Briefly, bacterial suspensions were heat inactivated at 90°C for 45 min and then washed to remove secreted proteins.

For *in vivo* challenge studies, bacterial suspensions were prepared in PBS and adjusted to 5 × 10^9^ CFU/ml by measurement of the optical density at 600 nm (OD_600_).

### Protein purification.

Recombinant ClfA N123 (amino acids 40 to 559) (30) and ClfA N23 (amino acids 220 to 559) proteins (30), SdrC (amino acids 182 to 496) (31), and ClfB (amino acids 44 to 542) (32) were cloned and expressed from genomic DNA of S. aureus strain Newman and purified from Escherichia coli by Ni^2+^ affinity chromatography, as previously described (33). GST-tagged ClfA N1 (amino acids 40 to 228) was purified on a GSTrap FF purification column (GE Healthcare). Endotoxin was removed from all the proteins using Detoxi-Gel endotoxin-removing columns (Thermo Scientific).

### Isolation and stimulation of human T cells.

Anonymized buffy coats were obtained from healthy blood donors at the Irish Blood Transfusion Service, Dublin, Ireland. Peripheral blood mononuclear cells (PBMCs) were isolated, and CD4^+^ cells were purified (CD4^+^ T cell isolation kit; Miltenyi Biotec) and labeled with carboxyfluorescein diacetate succinimidyl ester (CFSE) as previously described (5). PBMCs were gamma irradiated at 30 Gy with a ^137^Cs source (Gammacell 3000 Best Theratronics). CFSE-labeled CD4^+^ cells (1 × 10^5^) were cocultured with irradiated PBMCs (APCs) (1 × 10^5^) with cRPMI alone (negative control), staphylococcal enterotoxin A (100 ng/ml [positive control]), heat-inactivated S. aureus (1 μg/ml ≈ 1 × 10^7^ CFU/ml), or purified staphylococcal antigens (0.88 μM). cRPMI comprised RPMI (Sigma), 10% (vol/vol) fetal calf serum (Biosera), 100 mM l-glutamine (Gibco), and 100 μg/ml penicillin-streptomycin (Gibco). T cell proliferation and cytokine production were assessed on day 10 by flow cytometry.

### Murine immunization model.

Naive mice were vaccinated via subcutaneous (s.c.) injection with 50 μg/mouse CpG (Hycult Biotech) alone; CpG in combination with ClfA N123, N23, N1, or GST (1 μg/mouse); or vehicle (PBS) on days 0, 14, and 28. On day 63, the mice were challenged with S. aureus PS80 (5 × 10^8^ CFU) via intraperitoneal (i.p.) injection. Prior to challenge, ILN cells were collected for antigen recall and blood samples were collected for analysis of antibody titers. At 72 h postinfection, peritoneal exudate cells (PEC) were isolated by lavage of the peritoneal cavity and analyzed by flow cytometry. Whole kidneys and liver were homogenized in PBS. The bacterial burden was established by plating serial dilutions of the peritoneal lavage fluid or tissue homogenate on TSA.

### Flow cytometry. (i) Human experiments.

Brefeldin A (5 μg/ml; Sigma) was added to the CD4^+^ T cell-APC cocultures for the final 16 h of culture. The cells were stained for viability with Aqua amine-reactive dye (Invitrogen) before surface staining with fluorochrome-conjugated antibody against CD4. The cells were fixed and permeabilized using the Dako IntraStain fixation and permeabilization kit, followed by intracellular staining with fluorochrome-conjugated antibodies against IL-17A, IFN-γ, and TNF-α.

### (ii) Murine experiments.

Isolated PEC were incubated in the presence of phorbol myristate acetate (PMA) (50 ng/ml), ionomycin (500 ng/ml), and brefeldin A (5 μg/ml) for 4 h at 37°C before surface staining with fluorochrome-conjugated antibodies against CD3, CD4, CD8, and CD11b. The cells were fixed and permeabilized, followed by intracellular staining with fluorochrome-conjugated antibodies against IL-17A and IFN-γ. To investigate ROS activity within phagocytes, cells were stained with fluorochrome-conjugated antibodies against CD11b, F4/80, and Ly6G, and 123-dihydrorhodamine assays were carried out as previously described (34).

Flow cytometric data were acquired with a BD LSR Fortessa or BD FACSCanto II and analyzed using FlowJo software (Tree Star, Inc.).

### Measuring the cellular and humoral responses to antigen.

Lymphocytes were isolated from the ILN on day 63 postimmunization and restimulated *in vitro* with ClfA N123 (5 μg/ml) for 72 h. R&D Duo-set enzyme-linked immunosorbent assays (ELISAs) were used to determine IFN-γ (2-pg/ml limit of detection) and IL-17 (15.6-pg/ml limit of detection) secretion by ILN cells. ClfA-specific IgG antibody titers in sera were quantified by sandwich ELISA using anti-mouse IgG (1 in 4,000; Sigma-Aldrich), as previously described (35). Sera from CpG- plus N123-, N23-, and N1-vaccinated groups were diluted 1:400 in PBS, while sera from control groups (PBS and CpG) were diluted 1:2. Antibody concentrations were expressed as endpoint titers calculated by regression of a curve of OD values versus reciprocal serum levels to a cutoff point of 2 standard deviations above control serum. The presence of ClfA-specific neutralizing antibodies was determined by measuring the ability of serum (1:60 dilution) to inhibit the binding of S. aureus PS80 to fibrinogen via ClfA, as previously described (36).

### Statistical analysis.

Statistical analyses were performed using GraphPad Prism software. Differences between nonnormally distributed groups were analyzed using a Mann-Whitney U test or Kruskal-Wallis test with Dunn's multiple-comparison posttest. Differences between normally distributed groups were analyzed using one-way analysis of variance (ANOVA) with a Bonferroni posttest or two-way ANOVA with Tukey correction. A *P* value of <0.05 was considered significant.

## Supplementary Material

Supplemental material
