# Supplementary material for: The Staphylococcus aureus Cell Wall-Anchored Protein Clumping Factor A Is an Important T Cell Antigen
Source: Infect Immun. 2017 Nov 17;85(12):e00549-17. doi: 10.1128/IAI.00549-17 (PMC5695125; doi:10.1128/IAI.00549-17)
Supplement: Supplemental material [file supp_85_12_e00549-17__index.html]

Supplemental material 

# The Staphylococcus aureus Cell Wall-Anchored Protein Clumping Factor A Is an Important T Cell Antigen

## Supplemental material

- Supplemental file 1 -

  Table S1. Percentage of donors that responded to antigen stimulation. Fig. S1. No difference in levels of proliferation of human CD4+ T cells in response to media or bovine serum albumin. Fig. S2. Stability of subdomains of clumping factor A. Fig. S3. 72.9% of individual’s CD4+ T cells proliferate in response to purified ClfA N123. Fig. S4. Immunization with individual subdomains of ClfA plus CpG had no significant effect on the production IFN-γ or IL-17 by γδ T cells during *S. aureus* infection. Fig. Immunization with individual subdomains of ClfA plus CpG does not increase phagocyte recruitment to the peritoneal cavity. Fig. S6. No difference in levels of proliferation of human CD4+ T cells in response to heat-inactivated *S. aureus* LAC or LAC::*lux*.

  PDF, 243K
