# Supplementary material for: The Staphylococcus aureus Cell Wall-Anchored Protein Clumping Factor A Is an Important T Cell Antigen
Source: Infect Immun. 2017 Nov 17;85(12):e00549-17. doi: 10.1128/IAI.00549-17 (PMC5695125; doi:10.1128/IAI.00549-17)
Supplement: Supplemental material [file IAI.00549-17_zii012172228s1.pdf]

**TABLE S1. The percentage of donors that responded to antigens stimulation.** Donors with antigen-specific proliferation >0 % were designated a responder to the relevant antigen.

| **Antigen** | **% of Responders** | **p-value[a]** |
|---|---|---|
| HI LAC::*lux* WT | 90 % | |
| HI LAC::*lux srtA* | 77.5 % | p=0.224 vs. HI LAC::lux WT |
| ClfA N123 | 72.9 % | |
| ClfA N23 | 62.5 % | p=0.421 vs. ClfA N123 |
| ClfA N1 | 42.9 % | p=0.0283 vs. ClfA N123 |
| ClfB | 40 % | p=0.0034 vs. ClfA N123 |
| SdrC | 57.7 % | p=0.202 vs. ClfA N123 |

[a] *P* values were calculated using Fisher's exact test

HI=Heat-inactivated

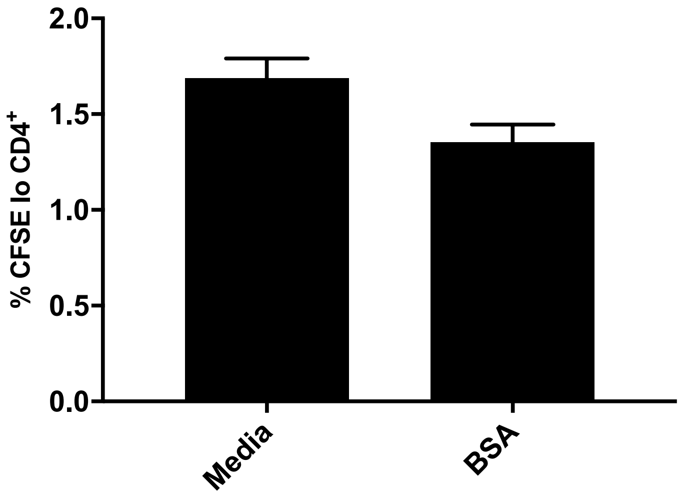


**FIG S1. No difference in levels of proliferation of human CD4+ T cells in response to media or bovine serum albumin.** Human PBMCs from healthy blood donors were CFSE-labelled and incubated with media alone or bovine serum albumin (1$\mu$g/ml). On day 10, proliferation was assessed by gating on $CFSE_{lo}$ cells in the $CD4^+$ population. Results expressed as mean ± SEM. n=3 per group.

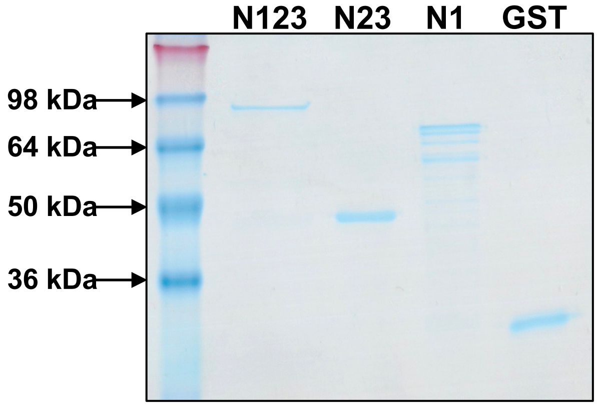


**FIG. S2. Stability of subdomains of clumping factor A.** ClfA N123, ClfA N23, ClfA N1 and GST were run on a 12 % SDS-PAGE gel and stained with Instant Blue (Expedeon). Slight breakdown of the ClfA N1 subdomain is observed.

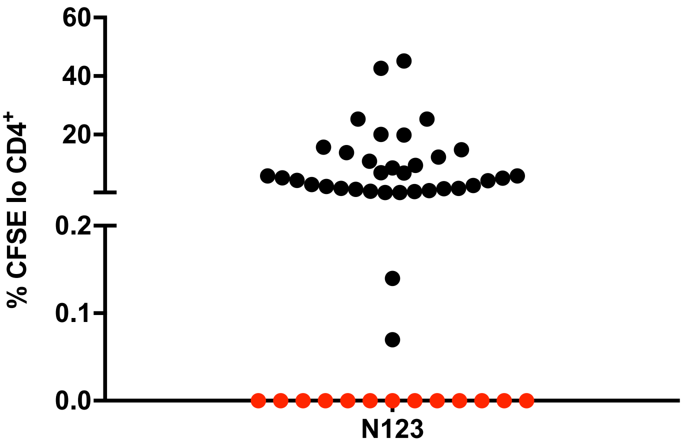


**FIG S3. 72.9 % of individual's CD4$^+$ T cells proliferate in response to purified ClfA N123.** Purified CD4$^+$ T cells and irradiated APCs from healthy blood donor buffy coats were CFSE-labelled and co-cultured with ClfA N123 (0.88$\mu$M) or media alone. After 10 d, proliferation was assessed by gating on CFSE$_{lo}$ cells in the CD4$^+$ population. Donor cells which failed to respond to ClfA N123 stimulation (indicated as red dots) were excluded from further study. n=48.

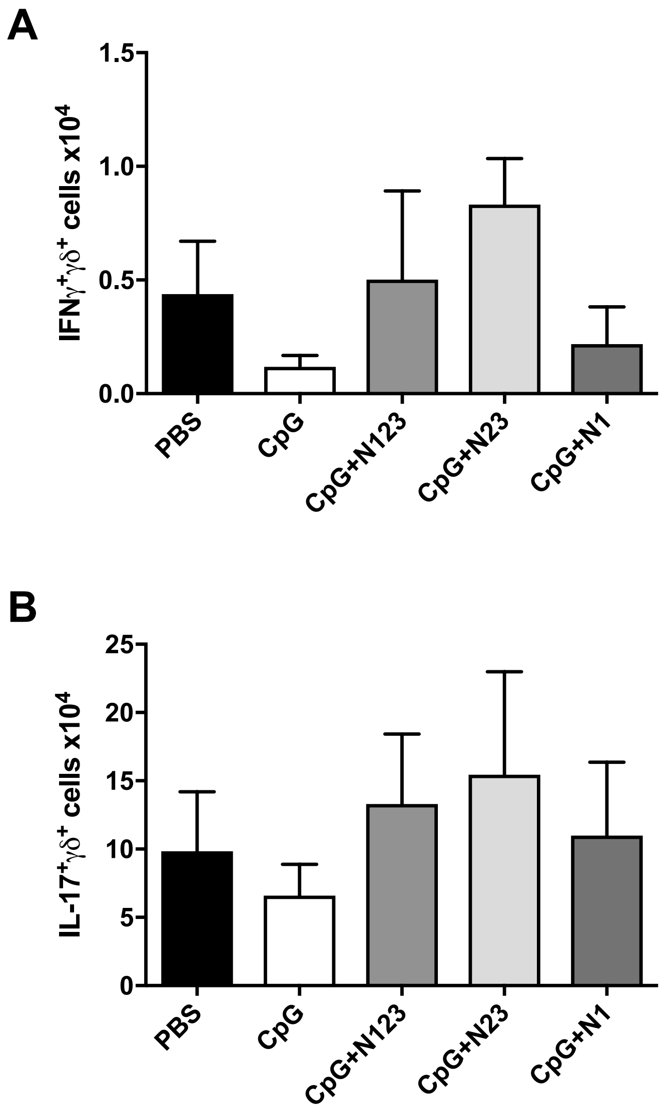


**FIG S4. Immunization with individual subdomains of ClfA+CpG had no significant effect on the production IFN$\gamma$ or IL-17 by $\gamma\delta$ T cells during *S. aureus* infection.** Mice were vaccinated with CpG (50$\mu$g/mouse)+ClfA N123, N23, N1 (1$\mu$g/mouse) via s.c. injection on d 0, 14, 28. On d 63 mice were challenged with *S. aureus* PS80 ($5x10^8$ CFU) via i.p. injection alongside a control group of sham-immunised (with PBS) mice. At 72 h post-infection, cells were isolated from the peritoneal cavity to assess absolute numbers of IFN$\gamma^+\gamma\delta^+$ (A) and IL-17$^+\gamma\delta^+$ (B) cells by flow cytometry. Results expressed as mean ± SEM. n=5 per group.

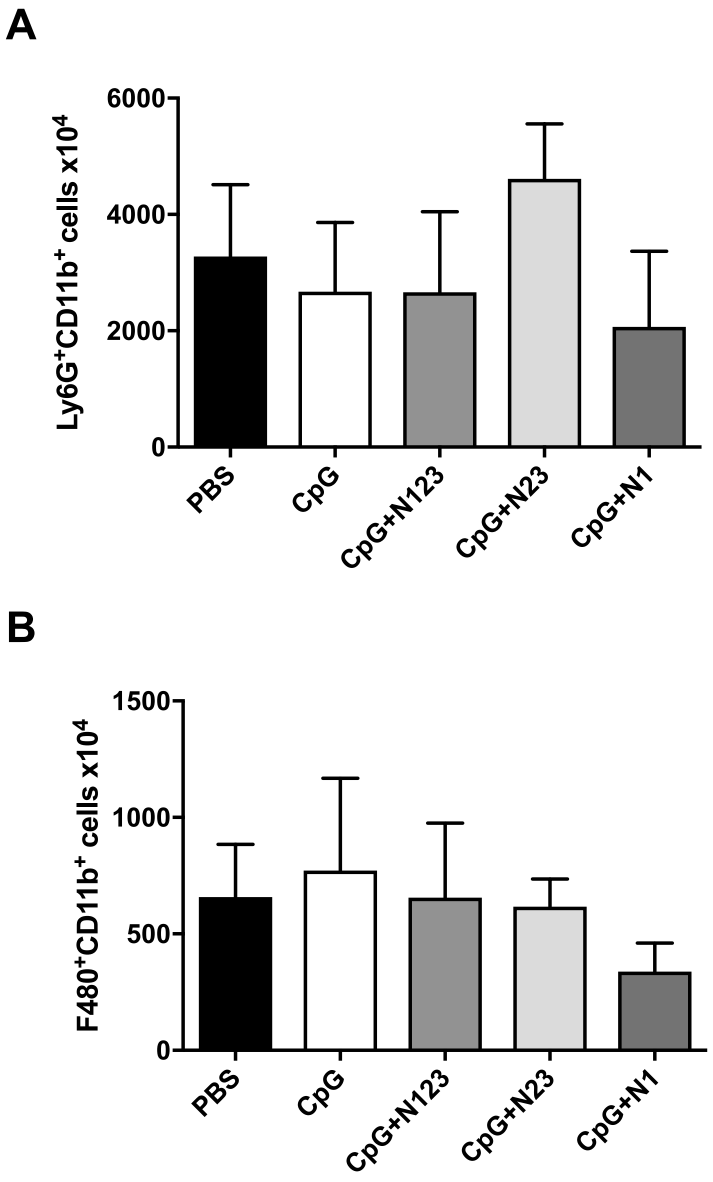


**FIG. S5. Immunization with individual subdomains of ClfA+CpG does not increase phagocyte recruitment to the peritoneal cavity.** Mice were vaccinated with CpG (50$\mu$ g/mouse)+ClfA N123, N23, N1 (1$\mu$g/mouse) via s.c. injection on d 0, 14, 28. On d 63 mice were challenged with *S. aureus* PS80 (5x$10^8$ CFU) via i.p. injection alongside a control group of sham-immunised (with PBS) mice. At 72 h post-infection the number of neutrophils [Ly6G$^+$F480$^-$] (A) and macrophages [F480$^+$Ly6G$^-$] (B) infiltrating the peritoneal cavity was assessed. Results expressed as mean ± SEM. n=5.

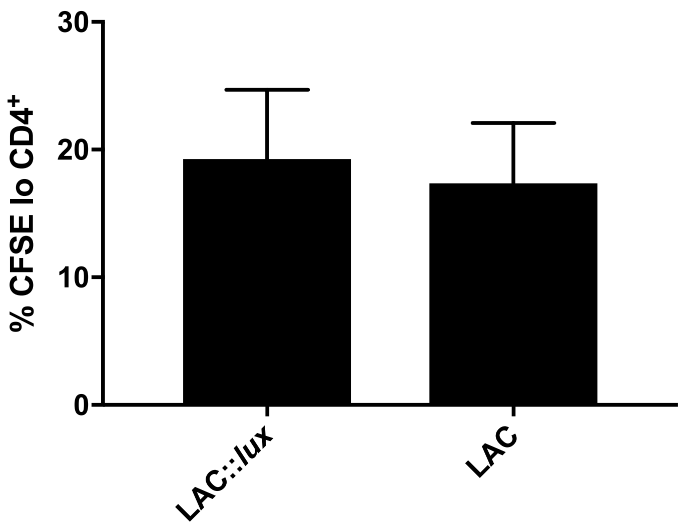


**FIG S6. No difference in levels of proliferation of human $CD4^+$ T cells in response to heat-inactivated *S. aureus* LAC or LAC::*lux*.** Human PBMCs from healthy blood donors were CFSE-labelled and incubated with heat-inactivated LAC or LAC::*lux* (1$\mu$ g/ml) or media alone. On day 10, proliferation was assessed by gating on $CFSE_{lo}$ cells in the $CD4^+$ population. For each patient, media only responses were subtracted from responses to heat-inactivated *S. aureus* to determine antigen-specific response. Results expressed as mean ± SEM. n=3 per group.
